# A new marine diatom (Bacillariophyceae) species – *Halamphoralombokensis* sp. nov. and the first observation for *H.banzuensis* from Kuta Beach Lombok, West Nusa Tenggara, Indonesia

**DOI:** 10.3897/phytokeys.250.132304

**Published:** 2024-12-27

**Authors:** Elya Putri Pane, Yenny Risjani, Paul B. Hamilton, Cüneyt Nadir Solak, Yunianta Yunianta, Nesil Ertorun, Elif Yılmaz

**Affiliations:** 1 Doctoral Program of Department of Aquatic Resources Management, Faculty of Fisheries and Marine Science, University of Brawijaya, 65145 Malang, East Java, Indonesia; 2 Aquaculture Master Program at Department of Aquatic Resources Management, Faculty of Fisheries and Marine Science, University of Brawijaya, 65145 Malang, East Java, Indonesia; 3 Research Center on Algae and Environment (ALGAEN Center), University of Brawijaya, Malang, Indonesia; 4 Research and Collections, Canadian Museum of Nature, P.O. Box 3443, Station D, Ottawa, Ontario K1P 6P4, Canada; 5 Department of Biology, Faculty of Science and Art, University of Dumlupınar, 43000 Kütahya, Turkiye; 6 Department of Food Science and Biotechnology, Faculty of Agricultural Technology, University of Brawijaya, 65145 Malang, East Java, Indonesia; 7 Department of Biology, Science Faculty, Eskişehir Technical University, 26000, Eskişehir, Turkiye; 8 Institute of Marine and Environmental Sciences, University of Szczecin, Szczecin, Mickiewicza 16A, PL70–383, Poland

**Keywords:** Diatoms, *H.Lombokensis*, *H.banzuensis*, Mandalika, Microalgae, new record, new species

## Abstract

This paper describes a new species of Halamphora – *Halamphoralombokensis***sp. nov.** and records for the first time *Halamphorabanzuensis* for the coasts of Indonesia. The study utilized light and scanning electron microscopy to meticulously examine the morphology. These species were found at Kuta Beach on the island of Lombok in Indonesia in highly saline sandy environments. The newly identified species, *H.lombokensis***sp. nov.**, is characterized by its semi-lanceolate to narrowly semi-elliptic shape with a straight ventral and convex dorsal margin. The study contributes to our understanding of marine diatom flora in Indonesia’s coastal regions.

## ﻿Introduction

Over the past decades, considerable effort has been expended to enhance our understanding of the diversity of marine and freshwater diatoms (Bacillariophyceae) in Indonesian aquatic ecosystems, starting with the classical work of Hustedt in 1943. In subsequent years, numerous new marine and freshwater diatom taxa were reported from islands throughout the Indonesian Ocean (Risjani et al. 2021), including those attributed to the *Luticola* D.G. Mann taxa from Maluku (Rybak et al. 2021, 2024), *Hantzschia* species from rural areas (Southeast Asia) (Rybak et al. 2022), *Achnanthidium* spp., from Lake Bratan, Bali (Kapustin et al. 2022), and from Temple Lake and Matano Lake (Tseplik et al. 2021a, b), *Catenula* species from Java and Sulawesi (Kryk et al. 2021), *Nupelabrevistriata* from soil and tree bark in Palambak and Sumatra (Rybak et al. 2019), *Gomphonemamatanensis* ancient Lake Matano (Kociolek et al. 2018), and 11 new taxa of Surirella from The Malili Lakes, Sulawesi (Bramburger et al. 2006). The richness of diatom taxa across Indonesia is well established with hotspots like the fossil deposits of Lake Toba (Sumatra) and the endemic assemblages of the Maili Lakes (Sulawesi) focal points for future investigations. Despite the existence of these discoveries, there is still a lack of understanding about littoral marine diatoms in Indonesia.

The genus *Halamphora*, a group of diatoms within the family Amphipleuraceae, has been extensively researched and revised over the past few decades. In addition to the classical literature on the subgenus Halamphora (Cleve 1895), Levkov (2009) studied an extensive number of *Amphora* species and officially elevated *Halamphora* to the level of genus. Stepanek and Kociolek (2013) have more recently made further extensive contributions to the discovery of marine *Halamphora* species using a combination of morphological examinations with electron and light microscopes and genetic analyses. *Halamphora* is distributed globally in freshwater and marine environments; there are 155 accepted species names and 8 accepted varieties (Algaebase). Genetically, *Halamphora* is monophyletic and positioned within the Family Amphipleuraceae next to the sister Rhopalodiaceae (Stepanek and Kociolek 2014). *Halamphora* is morphologically similar to the genus *Amphora* (Family Catenulaceae) but can be identified by distinct features, among which are the raphe ledge that exists only on the dorsal side in the genus *Halamphora*, while the areolae have round, elliptical, or transversely elongated shapes occluded by hymenes or vela and internally with small pores (Levkov 2009; An et al. 2022).

The reliability of identifying fine features in *Halamphora* species using light microscopy is enhanced by using scanning or transmission microscopy which is often essential for accurate identifications. The objective of this study is to describe a new species *Halamphoralombokensis* sp. nov. and record for the first time *Halamphorabanzuensis* for the coasts of Indonesia, Kuta Beach, West Nusa Tenggara, Lombok Island.

## ﻿Materials and methods

### ﻿Study area

**Figure 1.**
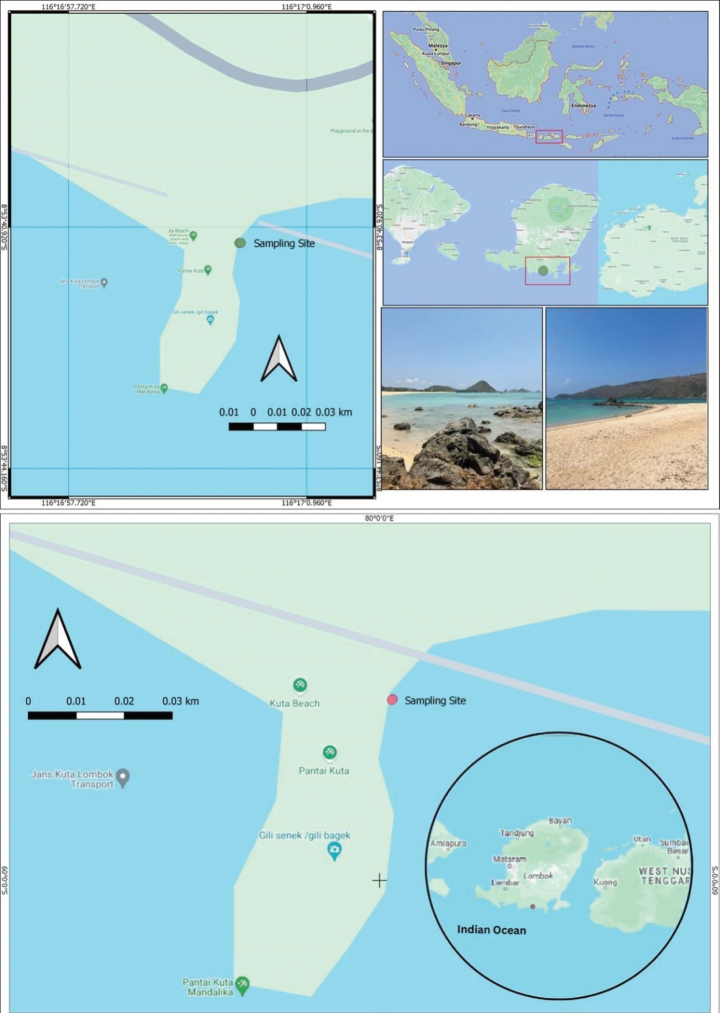
Sampling site of this study in Kuta Beach, Lombok Island, Indonesia.

Kuta Beach is one of many beaches on Lombok Island, located on the east side of the larger Bali Island (Fig. 1). The sampling station is located in Mandalika in the southern region of the Central Lombok District, West Nusa Tenggara Province with coordinates 8°53'41.132"S, 116°17'0.042"E. In contrast to other Indonesian beaches which collect extensive garbage and plastics, the coastal location with exposure to the Indian Ocean has clean and clear seawater. The beach is famous for its beautiful scenery and is a top choice for many tourists. This location is part of a unique eco-tourism management area, which includes the tourism zone, the open-access zone, and the support facilities near the Mandalika Circuit, an international-standard racing circuit. The beach is shaped like a bay and is approximately 3000 m long from west to east. It is bordered by hills and composed of white, ball-shaped sand from abraded coral reefs. The bathymetric in front of the beach is shallow across 2/3 of the eastern part, which can be dry during low tide, descending about 800 m from the beachline (Pradjoko et al. 2015).

### ﻿Sampling

Samples were collected in November 2023 from sandy and rocky substrata. The sampling sites along the seashore revealed seashore depths ranging from approximately two to five centimeters, clearly indicating relatively shallow substrate conditions. Epilithic samples were separated from the substrate using a toothbrush and epipelic samples were collected using a plastic pipette (Taylor et al. 2007). Rock substrates of varying random size and flatness were selected from Kuta Beach and thoroughly brushed clean over the andesitic, basalt, and granite stones. Water temperature (°C), dissolved oxygen (mg.L-1), oxygen saturation (%), pH, electrical conductance (µS.cm-1), and salinity (%) were measured in situ using a Lange Hach 40d multi-parameter meter. Samples were immediately transported back to the lab for study. The collected type materials are deposited at the Center for Algae and Environment (ALGAEN Center) at the Department of Aquatic Resources Management, Faculty of Fisheries and Marine Science, Brawijaya University, Malang, East Java, Indonesia. The holotype slide and material are deposited in the Canadian Museum of Nature (CANA), Ottawa, Ontario, Canada, and isotype slides in Kütahya Dumlupınar University (DPU), Türkiye.

### ﻿Sample preparation

Light (LM) and scanning electron microscope (SEM) materials were prepared by treatment with 10% HCl and 30% H2O2 to remove organic material following the method described in Swift (1967). To obtain permanent microscopic slides, cleaned material was mounted in Naphrax®. The slides were analyzed using a Nikon Ci Light microscope in the Diatom Laboratory at Kütahya Dumlupınar University (DPU), Türkiye. Observations were completed at 1000x magnification with a 100x Plan Apochromat oil immersion objective (NA = 1.4). The ultrastructure morphological observations were done using SEM. For that purpose, cleaned material was filtered through a polycarbonate membrane filter with a pore diameter of 5 μm. These membrane filters were fixed on aluminum stubs after air–drying. Stubs were sputter-coated with a gold layer reaching a thickness of ~20 nm and studied using a ZEISS Ultra field emission scanning electron microscope at the University of Eskişehir Technical University, Türkiye. Stubs of the type material are stored at the Center of Algae and Environment (ALGAEN Center), Faculty of Fisheries and Marine Science, University of Brawijaya, Malang, East Java, Indonesia, and Kütahya Dumlupınar University (DUP), Türkiye.

## ﻿Results

In this study, some environmental variables were measured during sampling in November 2023. The temperature was 33.3 °C, pH 7, salinity 32 ppt, and dissolved oxygen 18.5 mg.L^-1^.

**Figure 2.**
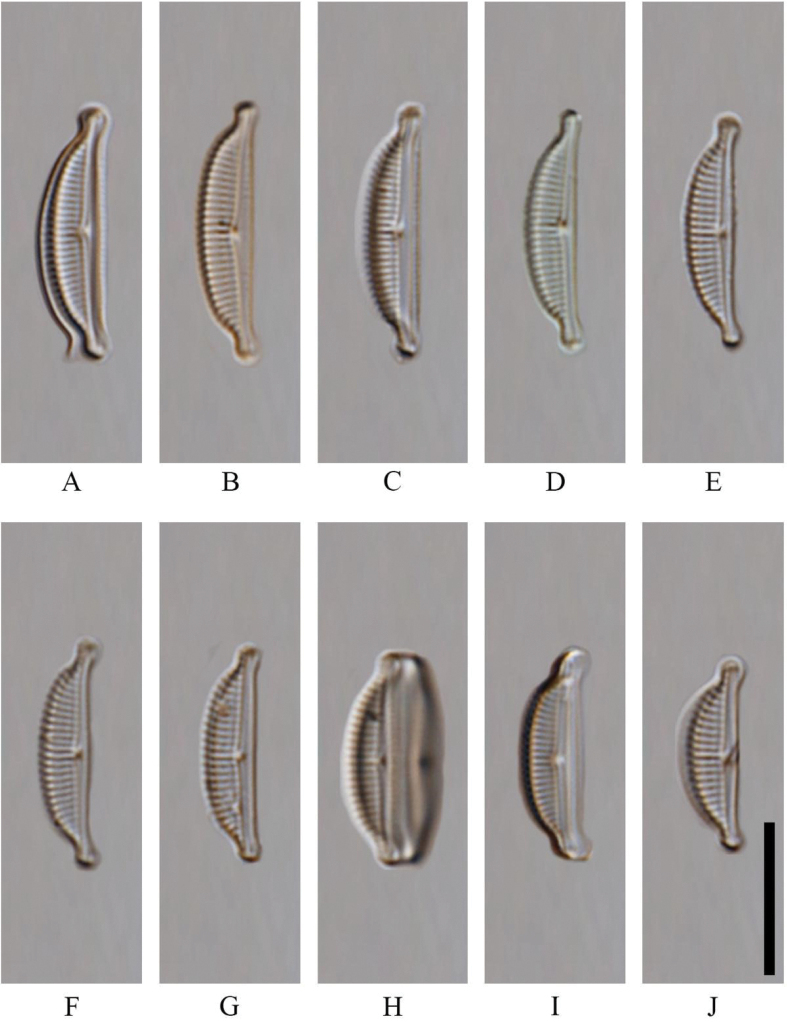
*Halamphorabanzuensis* from Lombok Kuta, Indonesia. LM micrographs of valves showing the size diminution series. Scale bar: 10 µm.

**Figure 3.**
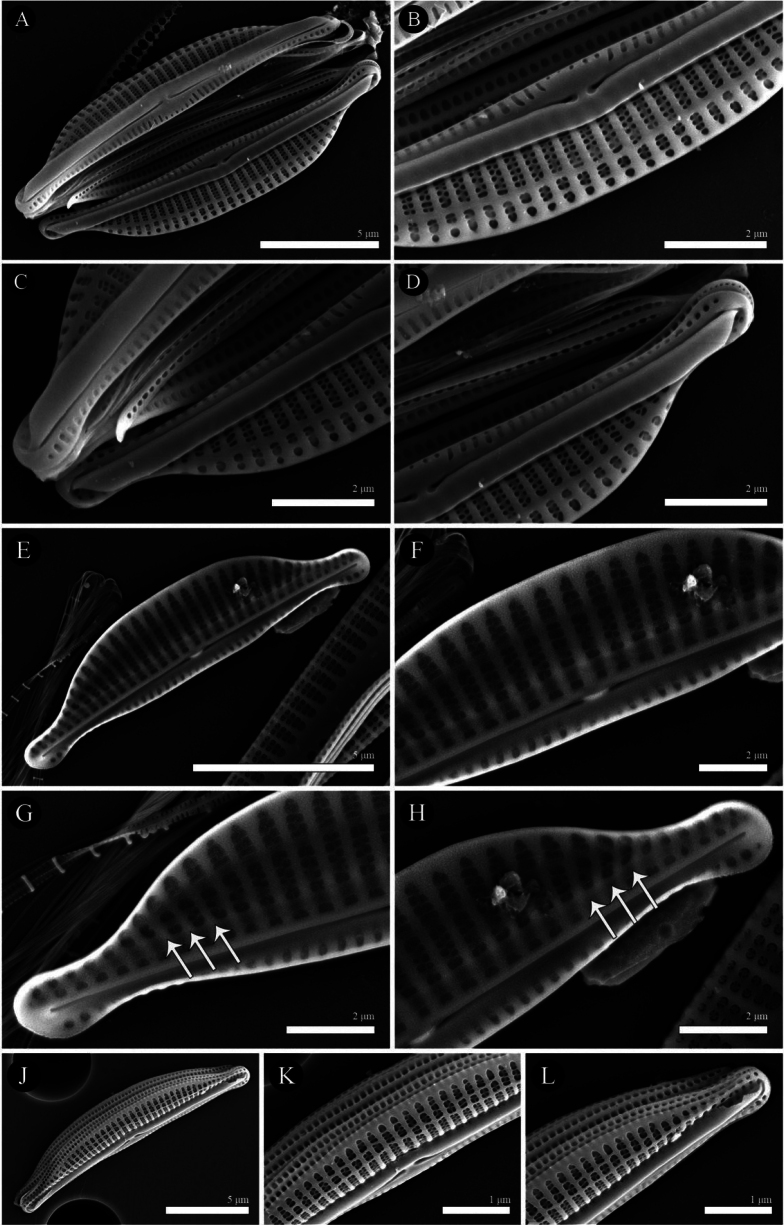
SEM micrographs of external view of *Halamphorabanzuensis***A** general external view of a valve **B** details of the central area showing proximal raphe endings **C, D** details of the apices showing distal raphe endings **E–H**SEM micrographs of internal view of *Halamphorabanzuensis***E** general internal view of a valve **F** details of the central area showing proximal raphe endings **G, H** details of the apices showing distal raphe endings and axial ridge. Arrows identify silica rib extending the length of the internal valve near the axial area on dorsal side. **J–L**SEM micrographs of external girdle view of *Halamphorabanzuensis***J** general external view of a valve **K** details of central area showing proximal raphe endings **L** details of the apices showing distal raphe endings. Scale bars: 5 μm (**A, E, J**); 2 μm (**B–D, F–H**); 1 μm (**K, L**).

Two species of seven *Halamphora* were present in large numbers from Kuta Beach. The frustules of *Halamphorabanzuensis* Stepanek, Mayama and Kociolek 2018: 73 (Figs 2, 3) are wedge-shaped with semi-elliptical valves showing subcapitate ends that deflect dorsally, 13.0–16.5 µm in length and 3.0–4.0 µm in width. The axial conopeum and raphe ledge are broad and even throughout and the striae are biseriate. A primary identifying feature in SEM for this species is a distinct silica rib extending the length of the internal valve near the axial area on the dorsal side (Fig. 2G, H). The other common species is new and here described below.

### ﻿Ochrophyta Cavalier-Smith, 1995

**Bacillariophyceae Haeckel, 1878**

**Thalassiophysales D.G. Mann, 1990**

**Catenulaceae Mereschkoewsky, 1902**

#### ﻿ Halamphora lombokensis

Taxon classificationPlantaeThalassiophysalesCatenulaceae

P.B.Hamilton, E.P.Pane, Y.Risjani & C.N.Solak sp. nov.

0D724C1A-C1AD-569A-93BF-AB669DC14BB1

##### Description (LM).

**Figure 4.**
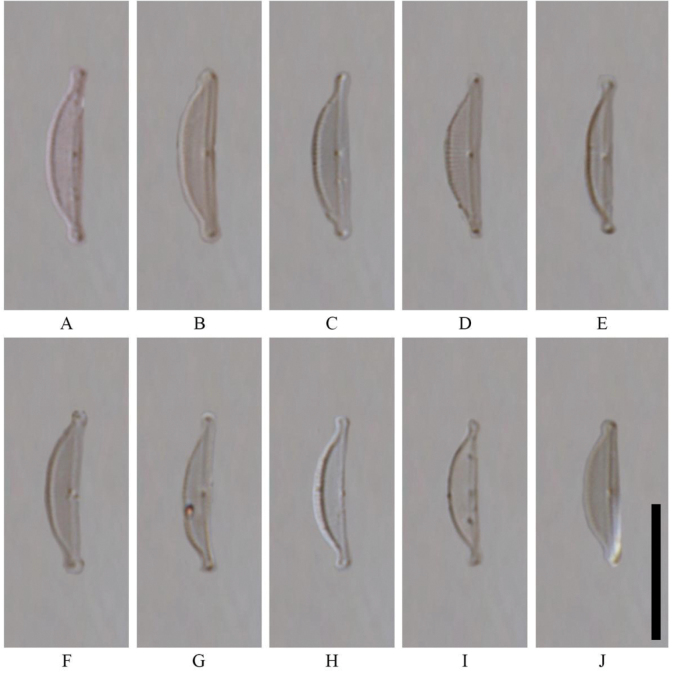
Type material of *Halamphoralombokensis* Hamilton, Pane, Risjani & Solak, sp. nov. from Lombok Kuta, Indonesia. LM micrographs of valves showing the size diminution series. Scale bar: 10 µm.

Valves semi-lanceolate to narrowly semi-elliptic with straight ventral and distinctively convex dorsal margins. Valve length 11.0–13.0 μm, breadth 2.0–3.0 μm. Valve ends slightly ventrally bent, rostrate to almost subcapitate. Raphe branches straight. Proximal raphe endings linear, distal raphe fissures not discernible in LM. The axial area narrow and the central area is almost absent. Striae is only visible under SEM. Dorsal striae density 28–32 in 10 µm (Fig. 4A–J).

##### SEM.

**Figure 5.**
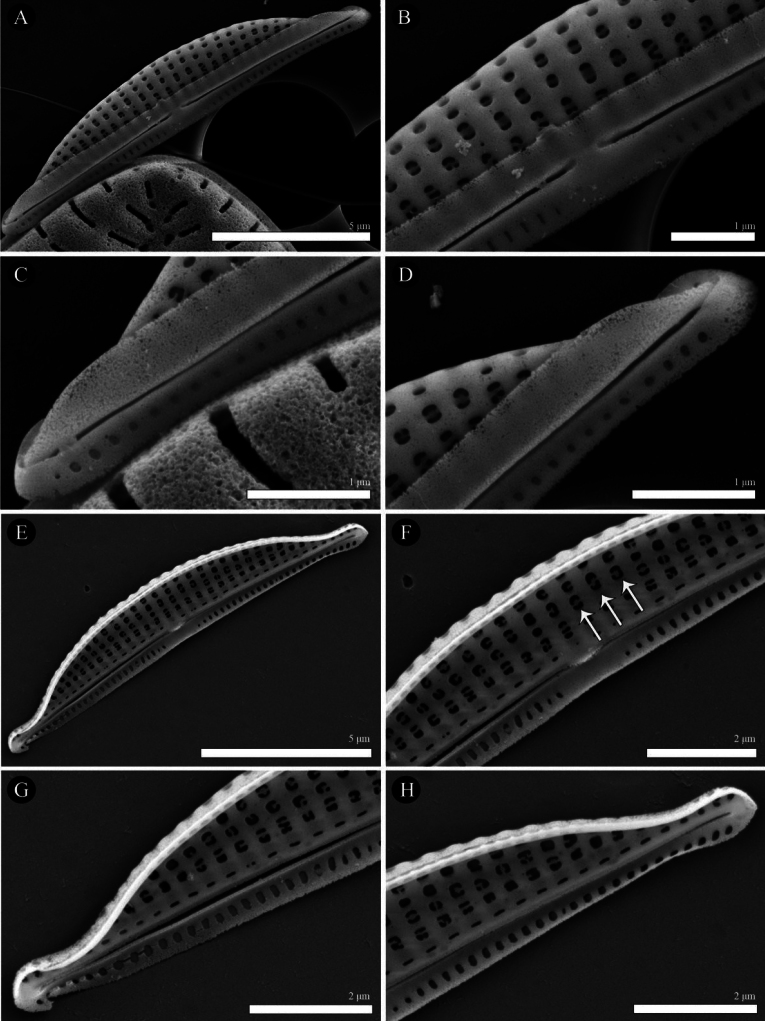
Type **A–D**SEM micrographs of external view of *Halamphoralombokensis* Hamilton, Pane, Risjani & Solak **A** general external view of a valve **B** details of central area showing simple depressed proximal raphe endings **C, D** details of the apices showing distal raphe endings. A small grove extends from the terminal raphe end towards the apex. **E–H**SEM micrographs of internal view of *Halamphoralombokensis***E** general external view of a valve **F** details of central area showing proximal raphe endings. Arrows highlight thick verminae separating the areolae. **G, H** details of the apices showing distal raphe endings. Scale bars: 5 μm (**A, E**); 2 μm (**F–H**); 1 μm (**B–D**).

Externally, valves are dorsiventral with narrowly convex dorsal margin and straight concave ventral margin. Central area absents on dorsal side and semi-lanceolate on ventral side. Raphe ledge extends from end to end, moderate, more or less equal width and weakly expanded towards the ends. Raphe branches weakly arched. Proximal raphe endings slightly expanded into straight central depressions. Distal raphe endings are hook-shaped. Dorsal striae are composed of uniseriate areolae; areolae elongated to become rounded towards margin. Areolae are composed of two elongated forms. Ventral striae with slit-like areola. Internally, areolae are round to elongate and divided into two pieces. Distal raphe endings terminating with poorly developed helictoglossae. Proximal raphe fissures fused into central helictoglossae. A distinct longitudinal band of silica runs from end to end near the dorsal axial area (Fig. 5A–H).

##### Material examined.

***Holotype***: slide number CANA 131860 (microscope slide designed as a holotype) in the collection of Nature Museum of Canada.

***Isotype***: Slide No. UB04 KUTA PSAL stored at the Center of Algae and Environment (ALGAEN Center) at the Faculty of Fisheries and Marine Science, University of Brawijaya, Malang, East Java, Indonesia • Slide no: INDO_Lombok Kuta_B2_(E.Pane)_Nov2023 observed at Kütahya Dumlupınar University Herbarium (DUP), Türkiye.

##### Type material.

Type material stored at the Center of Algae and Environment (ALGAEN Center), at Faculty of Fisheries and Marine Science, University of Brawijaya, Malang, East Java, Indonesia; subsample CANA 131860, analyzed at the Canadian Museum of Nature.

##### Type locality.

Indonesia, Kuta Beach, Lombok Island (GPS 8°53'41.132"S, 116°17'0.042"E), collector: Elya Putri PANE 11.11.2023.

##### Etymology.

Named for the Island of Lombok, where the species was found.

##### Distribution and ecology.

Observed from the type locality (Temperature 33.3 °C, pH 7.0, salinity 32 ppt. and dissolved oxygen 18.5 mg.L^-1^).

##### Differential diagnosis.

The species is similar to *Halamphoraangustiformis*Stepanek and Kociolek (2018: 68), *H.attenuata*Stepanek and Kociolek (2015: 32), *H.borealis* (Kützing) Levkov (2009: 175), *H.pellicula*Stepanek and Kociolek (2018: 50), *H.salinicola* Levkov & Díaz (Levkov 2009: 220), *H.tenuis*Stepanek and Kociolek (2018: 45) and *H.valdeminutissima*Zidarova et al. (2023: 202). Regarding striae density, *Halamphorapellicula*, *H.tenuis* and *H.valdeminutissima* have higher stria densities while the other comparable taxa have lower stria densities. Moreover, *H.angustiformis*, *H.attenuata* and *H.borealis* are larger while *H.pellucula* and *H.valdeminutissima* have smaller dimensions and typical long areolae. *Halamphoratenuis* is distinguished by biseriate areolae.

##### Associated diatom flora.

Unidentified species of another *Halamphora*, *Tabularia*, and *Navicula* were other frequent taxa in the sample.

## ﻿Discussion

**Figure 6.**
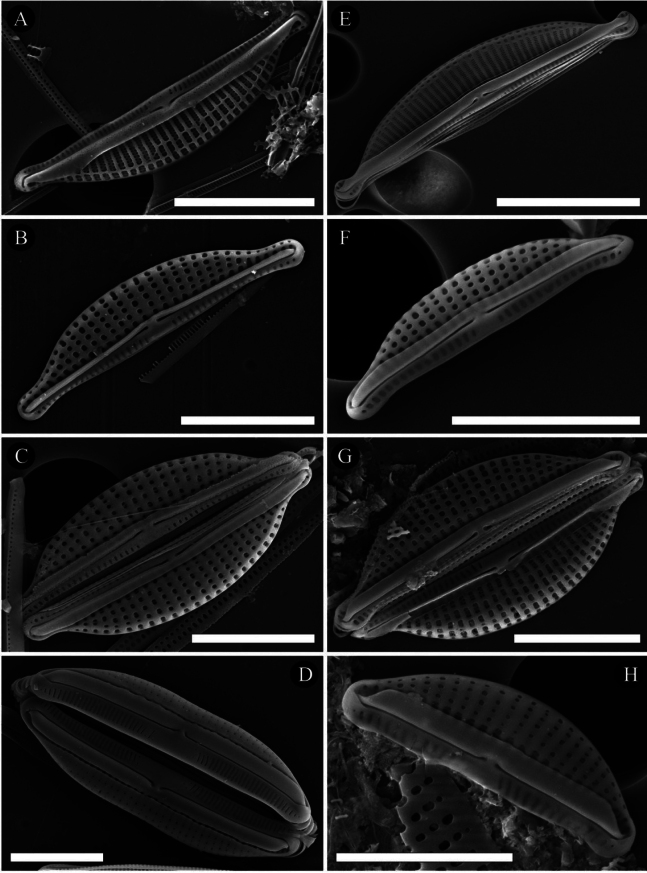
Five additional undescribed *Halamphora* taxa from Kuta Beach. A. Halamphoracf.hybrida. Scale bars: 10 μm.

The two species of *Halamphora* are identified from Kuta Beach on the island of Lombok Island, Indonesia; one is a new species. These marine taxa prefer highly saline epipsammic environments. The physicochemical results indicate marine coastal conditions with high temperature, high oxygen, and a circumneutral pH. The two species from the same microbiome are different in morphology and easy to distinguish through LM observations. Five additional poorly documented *Halamphora* taxa were abundant in Kuta beach (Fig. 6).

Taxonomically, these valve shapes (Fig. 6) are similar to a number of brackish and marine diatom taxa. Valve shape along with the formation of the raphe ledge, and striae and areolae (internal and external) are the primary characteristics that distinguish these *Halamphora* taxa. *Halamphoralombokensis* sp. nov., with a semi-lanceolate to narrowly semi-elliptic outline and capitate ends is a common valve outline. The new species is similar to the halophilous/brackish species (*H.salinicola*, *H.attenuata*), brackish species (*H.angustiformis*, *H.borealis*, *H.valdeminutissima*) and true marine species (*H.pellicula*, *H.tenuis*). *Halamphoraangustiformis*, like *H.lombokensis*, has a wide continuous dorsal raphe ledge and the areolae are uniseriate and unevenly distributed. *Halamphoraangustiformis* is distinguished by an expanded raphe ledge around the central area, deflected proximal and distal raphe ends toward the dorsal side, and larger valves with fewer striae. *Halamphoraattenuata* is a salt lake taxon from Dakota, USA (Stepanek and Kociolek 2015) and was identified by the large valves with a higher stria density, linear-elliptic dorsal valve margins, and protracted, not capitate ends. This saline lake taxon is distinguished by the higher density of areolae, differential formation of the areolae adjacent to the external dorsal axial area, and the absence of an internal silica rib next to the axial area. *Halamphoraborealis* was first described as *Amphoraborealis* by Kützing (1844: 108) and later changed to *Halamphora* by Levkov (2009). Distinctively, *H.lombokensis* and *H.borealis* have similar valve outlines, wide continuous raphe ledges from end to end, and irregularly spaced uniseriate dorsal areolae externally. Internally *H.borealis* has recessed biporoid pores between interrupted costae and continuous striae on the ventral side, whereas *H.lombokensis* has well-structured cribra over the areolae and interrupted striae on the ventral side at the central area. Also *H.borealis* has larger specimens (>19.0 µm length, >3.0 µm width) with a lower stria density (<25 in 10 µm). *Halamphorapellicula* was described from St. Joseph Bay in Florida, USA (Stepanek and Kociolek 2018). Valves of *H.pellicula*, like *H.lombokensis*, have a long, wide and continuous raphe ledge but the striae on the external valve face are not interrupted (uneven areolae distribution), there is a silica ridge at the valve face mantle junction (not in *H.lombokensis*) and the striae are finely biseriate. Internally, the striae are also finely biseriate between thickened costae, unlike the singular cribra covered areolae of *H.lombokensis*. *Halamphorapellicula* also has a higher striae density (>41 in 10 µm). *Halamphorasalinicola* was described from a salt lake in Atacama, Chile by Levkov and Díaz (Levkov 2009). This taxon has a broad continuous raphe ledge, and the central and dorsal raphe ends deflect dorsally. *H.salinicola* is distinguished from *H.lombokensis* by the biseriate poroid striae without cribra. The taxon is longer (>20.0 µm), has a lower stria density (<26 in 10 µm) and with shortly protracted and capitate endings. The marine *Halamphoratenuis* (Stepanek and Kociolek 2018) from Biscayne Bay Florida, USA is longer (>13.0 µm) and has a higher striae density (>32 in 10 µm). *Halamphoratenuis* is easily separated by the densely biseriate striae that are continuous across the valve face. *Halamphoravaldeminutissima* was described from Black Sea by Zidarova et al. (2023). The taxon has smaller specimens (<11.0 µm length, <3.0 µm width) and with a higher striae density (about 45 in 10 µm) (Table 1). This Black Sea taxon has a flat valve (curved in *H.lompokensis*), continuously biseriate striae with the marginal ridge sporadically interrupted by the striae. The secondary side of the central raphe ledge is also thickened.

**Table 1.** Morphological and meristic characteristics of *Halamphoralombokensis* (Hamilton, Pane, Risjani & Solak), sp. nov., and other *Halamphora* taxa sharing similar morphological features.

	Species valve length (µm)	Valve width (µm)	Dorsal stria density (in 10 µm)	Valve ending	Raphe ledge	Axial area	References
***Halamphoralombokensis*** Hamilton, Pane, Risjani & Solak sp. nov.	**11.0–13.0**	**2.0–3.0**	**28–32**	**protracted, narrowly rounded to subcapitate**	**broad, and linear**	**narrow with ventral fasica**	**this study**
***Halamphorabanzuensis*** (this study)	**13.0–16.5**	**3.0–4.0**	**22–23**	**protracted, narrowly rounded to subcapitate**	**broad, slightly dorsally elevated at centre**	**narrow dorsally and difficult to distinguish along the ventral side**	**this study**
*H.banzuensis* Stepanek, Mayama & Kociolek	16.0–17.0	3.0–3.5	20–22	protracted, narrowly rounded to subcapitate	broad, linear	narrow dorsally and difficult to distinguish along the ventral side	Stepanek and Kociolek 2018
*Halamphoraacutiuscula* (Kützing) Levkov	27.0–40.0	5.0–7.5	15–18	protracted, capitate	broad, linear, elevated dorsally	narrow throughout, widening ventrally	Levkov 2009
*Halamphoraangustiformis* Stepanek & Kociolek	15.0–33.0	3.0–4.0	20–22	protracted, narrowly rounded to subcapitate	broad, elevated dorsally	narrow throughout, widening ventrally	Stepanek and Kociolek 2018
*H.aponina* (Kützing) Levkov	23.0–40.0	3.0–4.5	20–22	protracted, capitate	narrow linear	narrow, widening ventrally	Levkov 2009
*H.attenuata* Stepanek & Kociolek	20.0–33.0	3.5–4.5	23–25	protracted, narrowly rounded	broad linear	narrow throughout, expending slightly ventrally	Stepanek and Kociolek 2015
*H.banzuensis* Stepanek, Mayama & Kociolek	16.0–17.0	3.0–3.5	20–22	protracted, narrowly rounded to subcapitate	broad, linear	narrow dorsally and difficult to distinguish along the ventral side	Stepanek and Kociolek 2018
*H.borealis* (Kützing) Levkov	19.0–40.0	3.0–4.0	20–24	protracted, capitate	narrow linear	narrow, widening ventrally	Levkov 2009
*H.crenulatoides* Stepanek & Kociolek	16.0–23.0	3.0–4.0	17–23	protracted, subcapitate	broad, linear	narrow dorsally and ventrally expanded	Stepanek and Kociolek 2018
*H.nagumoi* Stepanek, Mayama & Kociolek	16.0–24.0	3.0–3.5	18–20	weakly protracted, narrow, rounded	very broad, linear	narrow dorsally and ventrally expanded	Stepanek and Kociolek 2018
*H.pellicula* Stepanek & Kociolek	10.0–18.0	2.5–3.5	41–42	protracted, subcapitate	broad, linear	not well defined	Stepanek and Kociolek 2018
*H.salinicola* Levkov & Díaz	20.0–34.0	2.5–3.7	21–26	shortly protracted and capitate	narrow, expanded on both valve sides	narrow, widening ventrally	Levkov 2009
*H.tenuis* Stepanek & Kociolek	13.0–19.0	2.5–3.0	31–32	protracted, subcapitate	moderate, linear	narrow throughout	Stepanek and Kociolek 2018
*H.valdeminutissima* Zidarova et al.	6.0–13.5	1.5–3.0	about 45	protracted, subcapitate	narrow, slightly widening at centre and apices	narrow, widening ventrally	Zidarova et al. 2022

*Halamphorabanzuensis* from Banzu Flats in Tokyo Bay, Japan (Stepanek and Kociolek 2018) is geographically close and environmentally similar to the Kuta Beach assemblage, documenting a range extension for this species from 35 to 8°N which is a broad range extension from the East China Sea to the Timor Sea. The new documented range for this taxon is additional evidence that marine *Halamphora* species in the Indo-Pacific can have wide geographic distributions. It is worth noting that the population from Banzu flats is marginally larger and the dorsal side of the valve is continuously arched, while the Kuta population has more flattened dorsal valve margins.

Although the valve outlines of these two taxa from Kuta beach are common (linear-elliptic dorsal margin to more broadly rounded valve margin with protracted rostrate to capitate apices), fine structural features of the areolae, raphe ledge development and formation of silica ridges (or partial ridges) help distinct these species. Taxa with uniseriate taxa, like *H.lombokensis*, have a diverse assemblage of areolae structure, while species with recessed biseriate striae are less morphologically diverse with continuous or disrupted areolae along the striae. At present, species from inland saline waters, brackish waters and marine waters appear to be taxonomically separated.

Other new species of the genus Halamphora have been described recently, for example *Halamphoraminima*, new brackish diatom species from the mudflat of Korea (An et al. 2022) and two species from Livingstone Islands, Antarctica (Zidarova et al. 2022). The new and rare species from Lombok, Indonesia contribute to the new records of species from marine tropical regions. Kuta beach is a strict marine environment and the extended geographical distribution of *H.banzuensis* suggests that marine *Halamphora* species in Asian waters likely have broad distributions.

## Supplementary Material

XML Treatment for Halamphora lombokensis

## ﻿Additional information

### Author ORCIDs

Elya Putri Pane https://orcid.org/0009-0007-2678-0665

Yenny Risjani https://orcid.org/0000-0002-6191-5824

Paul B. Hamilton https://orcid.org/0000-0001-6938-6341

Cüneyt Nadir Solak https://orcid.org/0000-0003-2334-4271

Yunianta Yunianta https://orcid.org/0000-0002-9684-8131

Nesil Ertorun https://orcid.org/0000-0001-6224-7314

Elif Yılmaz https://orcid.org/0000-0001-7939-1814

### Data availability

All of the data that support the findings of this study are available in the main text.
